# Towards integrative gene functional similarity measurement

**DOI:** 10.1186/1471-2105-15-S2-S5

**Published:** 2014-01-24

**Authors:** Jiajie Peng, Yadong Wang, Jin Chen

**Affiliations:** 1 School of Computer Science and Technology, Harbin Institute of Technology, Harbin, China; 2 MSU-DOE Plant Research Laboratory, Michigan State University, East Lansing, MI 48824, USA; 3 Department of Computer Science and Engineering, Michigan State University, East Lansing, MI 48824, USA

**Keywords:** Gene ontology, Semantic similarity, Integrative measure

## Abstract

**Background:**

In Gene Ontology, the "Molecular Function" (MF) categorization is a widely used knowledge framework for gene function comparison and prediction. Its structure and annotation provide a convenient way to compare gene functional similarities at the molecular level. The existing gene similarity measures, however, solely rely on one or few aspects of MF without utilizing all the rich information available including structure, annotation, common terms, lowest common parents.

**Results:**

We introduce a rank-based gene semantic similarity measure called InteGO by synergistically integrating the state-of-the-art gene-to-gene similarity measures. By integrating three GO based seed measures, InteGO significantly improves the performance by about two-fold in all the three species studied (yeast, *Arabidopsis *and human).

**Conclusions:**

InteGO is a systematic and novel method to study gene functional associations. The software and description are available at http://www.msu.edu/~jinchen/InteGO.

## Background

The Gene Ontology (GO) provides a structured, controlled vocabulary of terms, which are interrelated forming a directed acyclic graph (DAG) for describing and categorizing (into three categories) the attributes for genes, gene products and sequences [1]. The "molecular function" (MF) category describes fundamental biochemical activities (including specific binding to ligands or structures of a gene product) at the molecular level [2]. As a popular resource used for functional annotation, MF provides rich information and a convenient way to study gene functional similarity by comparing terms with which the genes are annotated [3-7], which subsequently supports a wide variety of applications, such as assessing target gene functions [8], predicting gene functional associations [9], inferring protein nomenclature [10], predicting sub-cellular localization [11], discovering new pathways [12], *etc*.

In order to compute gene-to-gene functional similarities using GO, various computational approaches have been developed. These approaches can be classified into two distinct categories: 1) group-wise, meaning calculating gene-to-gene similarity directly based on a statistical framework considering all the terms annotated to the target genes [13-15], and 2) pair-wise, *i.e*., indirectly computing gene-to-gene similarity using term-to-term similarities computed with GO semantic measures [12,16-21]. Each of the aforementioned measurements adopts one or a few kinds of knowledge in the GO efficiently. However, they do not rely on all of the rich information available in the GO databases. In this paper, we propose a new rank-based gene semantic similarity measure called InteGO (Integrated Gene Ontology measure), which can integrate the state-of-the-art gene-to-gene measures [12,13,17] (therefore considering more information than these measures) to bring the performance of the GO-based functional similarity studies to a higher level.

**Figure 1:**
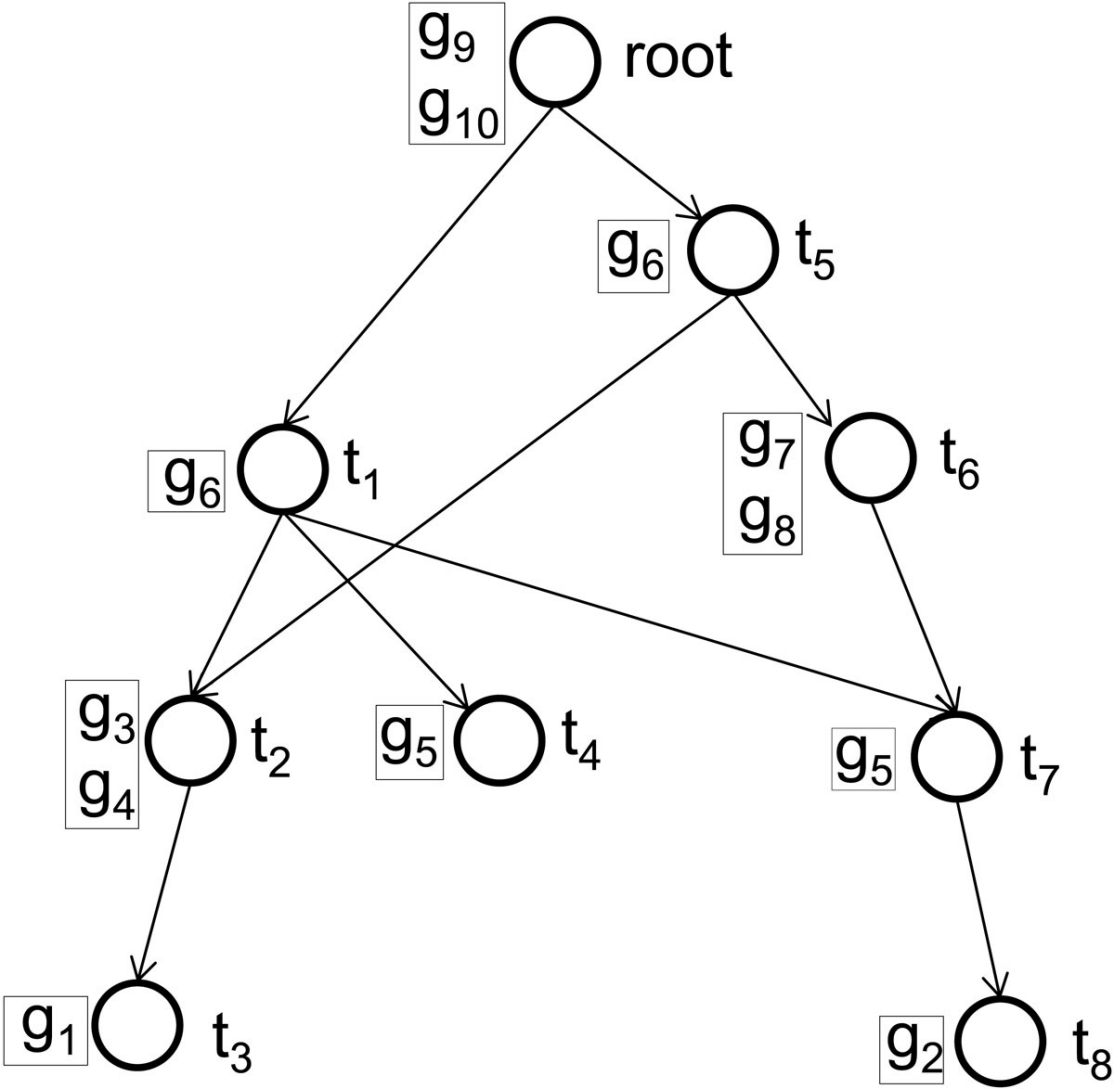
**An illustrative example of Gene Ontology (GO)**. An illustrative example of GO forming a directed acyclic graph (DAG), in which nodes and edges represent GO terms and "is-a" or "part-of" relationships between terms. {*t*_1_, ..., *t*_7_, *root*} is the set of GO terms, and {*g*_1_, ..., *g*_10_} is the set of genes annotated to these terms.

In the first GO-based measure category (group-wise), by combining elements of the topology and annotation information, the Yu measure calculates a probabilistic level of similarity from GO, in order to directly compute gene similarity [13]. The main idea of the Yu measure is that a pair of genes should be very similar if they are included in a functional group with a few proteins, whereas the similarity is lower if the gene pair belongs to a large gene group. Mathematically, given two gene *g*1 and *g*2, the gene-to-gene similarity can be calculated with:

(1) $$GeneSim_{Yu}\left(g_{1},g_{2}\right)=-\text{ln}\frac{n_{g1,g2}}{N}$$

where *n*_*g*1,*g*2_is the total number of gene pairs that have the same set of lowest common ancestors (LCAs) as *g*_1 _and *g*_2_; *N *is the total number of gene pairs in the selected GO category. A LCA is the common ancestor with the highest information content (IC). In the illustrative example in Figure 1, there are in total 45 gene pairs possible among the ten genes; the LCA of gene pair *g*_1 _and *g*_2 _is *t*_1_, and the number of gene pairs (which LCA is also *t*_1_) is 9. Therefore, the similarity of *g*_1 _and *g*_2 _based on the Yu measure is *−ln*(9*/*45) = 1.61. The Yu measure considers both the elements of topological distance and the LCA distance. However, it simplifies the computation of shared information of both genes without using all of the common parents of the GO terms annotated to *g*_1 _or *g*_2_, which neglects the locations of LCAs and the aggregate semantic contributions from the parents of the target terms (due to the high complexity of graph matching). Alternatively, the SORA [15] measure computes the IC of a term set by means of combining inherited and extended information content of the terms based on the structure of GO. Gene functional similarity is estimated using the IC overlap ratio of term sets. However, like the Yu measure, it ignores valuable information implicit in the semantics, *i.e*., the common parents of the GO terms, when calculating the shared IC and relationships among involved terms.

In the measures in the second category (pair-wise), the pair-wise term comparisons originally developed for natural language processing [16,18-21] are utilized, and are strongly dependent on the specific taxonomy. Among the earlier developed methods, an IC based measure called the Resnik measure has showed strong correlations between its results and gene expression similarities on yeast [16,22]. Mathematically, given a GO term *t*, its IC is defined as a negative log likelihood *IC*(*t*) = *− *log(*|G_t_|/|G_root_|*), where *G_t _*and *G_root _*are the sets of genes annotated to term *t *and the root term (including all of its descendants) respectively. In the Resnik measure, the similarity between term *t*_1 _and *t*_2 _is defined as the IC of LCA: *TermSim_Resnik _*(*t*_1_, *t*_2_) = *IC*(*LCA*_12_). Although the Resnik measure strongly correlated with the gene expression data [22], terms sharing the same LCA have the same semantic similarity, even if they are at very different levels of GO. Consequently, it cannot differentiate term pairs that are far from LCA with term pairs close to the same LCA. In the illustrative example in Figure 1, the common parent of *t*_2 _and *t*_7 _is *t*_1_, which is the same as the LCA of *t*_3 _and *t*_8_. According to the Resnik measure, *Sim_Resnik _*(*t*_2_, *t*_7_) = *Sim_Resnik _*(*t*_3_, *t*_8_) = 0.51, but clearly the distance from *t*_2 _and *t*_7 _to the LCA is shorter. To take both the distance from LCA to the target terms and the distance from LCA to root into account [17], a later-developed measure called the Schlicker measure was proposed:

(2) $$TermSim_{Schliker}\left(t_{1},t_{2}\right)=\frac{2\times IC\left(LCA_{12}\right)}{IC\left(t_{1}\right)+IC\left(t_{2}\right)}\times \left(1-\frac{\left|G_{LCA_{12}}\right|}{\left|G_{root}\right|}\right)$$

where $G_{LCA_{12}}$ is the set of genes annotated to the LCA of *t*_1 _and *t*_2_.

In Eq 2, the first part on the right side of the equation quantifies the distance from terms *t*_1 _and *t*_2 _to their LCA, and the second part measures the distance from the LCA to the root, where a short former distance and a long later distance indicate a higher similarity. Experimental results revealed that the Schlicker measure agrees with sequence similarity [17]. In the same example in Figure 1, the Schlicker measure is able to differentiate term pair (*t*_2_, *t*_7_) and (*t*_3_, *t*_8_) with *TermSim_Schlicker_*(*t*_2_, *t*_7_) = 0.15 and *TermSim_Schlicker_*(*t*_3_, *t*_8_) = 0.09. However, the common problem of the Schlicker measure and the Resnik measure is that they only consider a single common ancestor, neglecting the fact that two GO terms may have multiple common ancestors in the GO structure [23].

Recently, the Wang measure was proposed to consider all of the parent terms of the target terms [12]. Given a term *t*_1 _and its parent term *p*, the semantic contribution of *p *to *t*_1_, denoted as *S*_*t*1,*p*_, is defined as the maximal semantic contribution of the paths from *t*_1 _to *p*. The GO term similarity in the Wang measure is defined in Eq 3, where *P*_1 _(or *P*_2_) are the sets of all of the parents of *t*_1 _(or *t*_2_).

(3) $$TermSim_{Wang}\left(t_{1},t_{2}\right)=\frac{\underset{p\in P_{1}\cap P_{2}}{\sum }\left(S_{t_{1,p}}+S_{t_{2,p}}\right)}{\underset{t\in P_{1}}{\sum }S_{t_{1,p}}+\underset{t\in P_{2}}{\sum }S_{t_{2,p}}}$$

The experiment result shows that this measure performs significantly better than Resnik measure on yeast protein functional similarities [12]. However, the Wang measure ignores both the topological distances among the LCAs and the statistics of gene annotations that the Yu measure has taken into consideration. For the same example in Figure 1, to compare the similarity of term *t*_3 _and *t*_8_, all of the common parents of the two terms, *P*_3 _= {*t*_1_, *t*_2_, *t*_3_, *t*_4_, *t*_5_, *root*} and *P*_8 _= {*t*_1_, *t*_5_, *t*_6_, *t*_7_, *t*_8_, *root*}, are considered by the Wang measure.

For the Resnik, Schlicker and Wang measures, gene-to-gene similarity is computed based on the GO term similarities that annotate to the target genes. In Wang *et al *[12], let *g*_1 _and *g*_2 _be two genes and *T*_1 _and *T*_2 _be the sets of GO terms annotated to *g*_1 _and *g*_2_, the gene-to-gene similarity is calculated by Eq 4:

(4) $$GeneSim\left(g_{1},g_{2}\right)=\frac{\underset{t\in T_{1}}{\sum }TermSim\left(t,T_{2}\right)+\underset{t\in T_{2}}{\sum }TermSim\left(t,T_{1}\right)}{\left|T_{1}|\;\text{ + }\;|T_{2}\right|}$$

where *t *is a GO term, *TermSim*(*t*, *T_x_*) = max_*ti∈Tx *_Sim(*t*, *t_i_*), which represents the highest similarity between *t *and term set *T_x_*. Note that, for both *|T*_1_*| *and *|T*_2_*|*, only the terms with *T ermSim*(*t*, *T_x_*) ≠ 0 are counted.

To the best of our knowledge, the existing measures emphasize on only one or few types of relationships between genes but ignores the others. One of these measures may be better than the others on one specific set of terms and genes, but may perform worse than the other measures on another gene set. Since none of the existing measures takes into account all of the aspects of GO (structure, annotation, LCA, all of the common parent, etc), which is of course a challenging task, it is hypothesized that the integration of multiple measures can improve the performance, since integration of multiple methods has been widely applied for performance boosting [24-26]. In this paper, we proposed a rank-based gene semantic similarity measure called InteGO by synergistically integrating the state-of-the-art gene-to-gene similarity measures. The integrated measures are called seed measures in the rest of paper. The major contributions of our work are:

• While the existing measures only consider one or few aspects of the problem, InteGO is an integrative approach, which conceptually considers all of the information in GO to reduce incorrect score assignments. In addition, InteGO employs an adaptive approach for the optimization of the seed measure integration.

• A rank-based approach is used to integrate multiple seed measures. Since the values from different seed measures have different scales and distributions, a direct integration of the values may lead to biased results. With our rank-based approach, InteGO unifies the scale and distribution among different seed measures, ensuring fair comparison.

• InteGO is an open framework, which adds the flexibility to integrate more GO similarity measures, more advanced evaluation and integration methods in the future.

InteGO was systematically tested on three species with different levels of complexity of GO annotations, i.e., yeast, *Arabidopsis *and human. The experimental results on all of the three species show that InteGO performs consistently better than the other measures in all of the tests.

## Method

In order to integrate multiple seed measures in InteGO, two key problems need to be solved: first, how to select the most appropriate seed measures for integration; second, how to integrate all of the scores from the different seed measures. To solve these problems, InteGO is divided into two steps: 1) to compute similarity scores with every seed measure individually and rank the scores, and 2) to evaluate and integrate the ranks of multiple seed measures.

### Rank-based similarity

The outputs of the different gene-to-gene similarity measures have different scales and distributions. Therefore, a direct integration of the values may lead to biased results. In InteGO, we unify the scale and distribution among different seed measures with a rank-based approach. One common problem of rankbased approaches though is the data size dependence, *i.e*., while a rank-based approach can work well on a relative large dataset, it is often inadequate on a small set of data. For example, if only two genes are provided by a user, the similarity rank of the two genes is always one, regardless how high or how low the actual similarity score is. Therefore, instead of requiring users to always provide a large set of genes to compare (which is not reasonable all of the time), InteGO maintains a background set of genes (*BG*) for every species of interest to unify the similarity scores from the multiple seed measures. *BG *must satisfy two requirements: 1) it is large enough; 2) it unbiasedly includes the full spectrum of gene similarity scores, ranging from the lowest to the highest.

**Figure 2:**
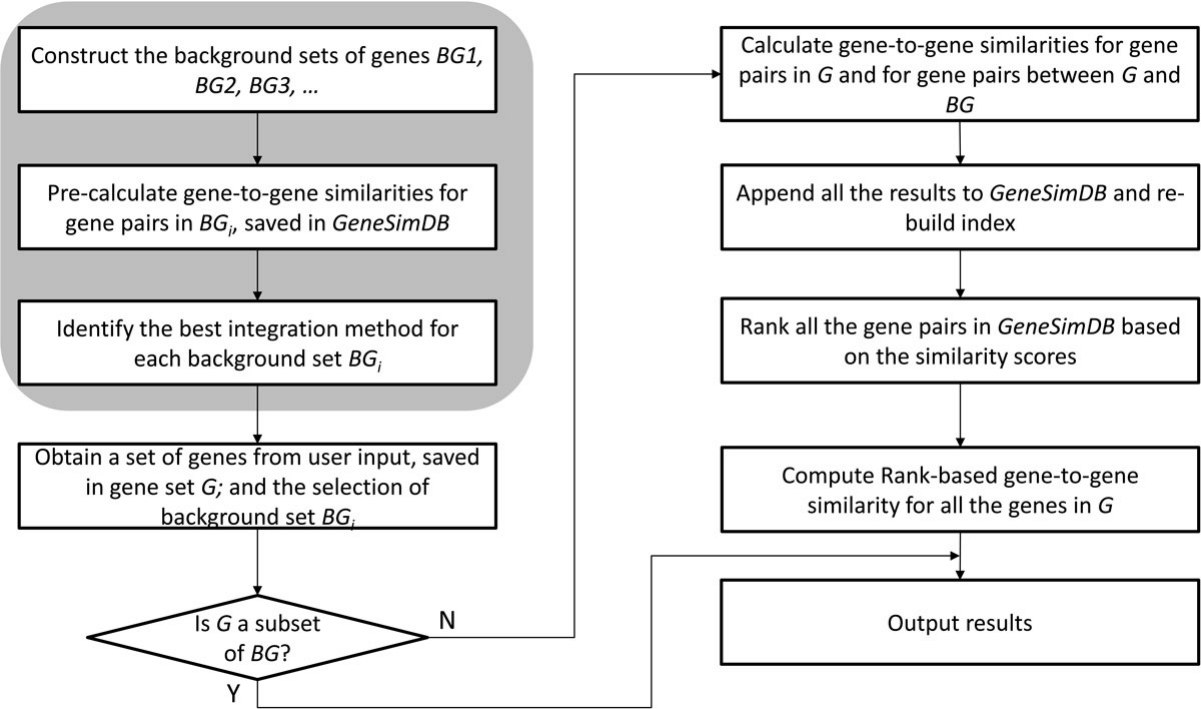
**Framework of InteGO for calculating the rank-based gene-to-gene similarities in MF**. Framework of InteGO for calculating the rank-based gene-to-gene similarities in MF. The boxes in the grey block are the pre-processing modules for the preparation of the background gene set.

The framework of InteGO is shown in Figure 2. In the steps with grey background, the similarity scores in *BG *are pre-calculated with all of the seed measures and saved in a database called *GeneSimDB*. When a user inputs a gene set *G*, the similarity scores of all of the gene pairs in *G *and all of the gene pairs between *G *and *BG *will be calculated with all of the seed measures, and be merged into *GeneSimDB*. If *G *is a subset of *BG*, InteGO will output the results directly. Finally, all of the gene pairs in *GeneSimDB *are sorted incrementally based on their gene similarity scores and are ranked. The ranked gene similarity score *RankSim*(*g*_1_, *g*_2_, *m*) for genes *g*_1 _and *g*_2 _in *G *is calculated as:

(5) $$RankSim\left(g_{1},g_{2},m\right)=\frac{2\times r_{g_{1},g_{2}}^{m}}{{\left(\left|BG\cup G\right|\right)}^{2}}$$

where $r_{g_{1},g_{2}}^{m}$ is the rank of gene pair *g*_1 _and *g*_2 _using seed measure *m*, and *BG *is the predefined background gene set, and *G *is the user provided gene set. The ranked similarity indicates how similar a given gene pair is in the background of all of the gene pairs.

One advantage to use the rank-based measure is to unify different scales and distributions among the seed measures. Therefore, the agreement among the ranks could indicate the functional similarities appropriately. An illustrative example is shown in Table 1. Given ten gene pairs, three measures (*M_A_*, *M_B _*and *M_C_*) are used to calculate the gene-to-gene semantic similarities based on the GO. The first column of the values show that the similarity scores of measure *M_A_*, *M_B _*and *M_C _*have different scales and different distributions. For example, the semantic similarity of gene pair 3 is 3.0 for measure *M_A _*and 0.9 for measure *M_B_*, although they both mean the highest functional similarity in their own datasets. The second column of the values show the ranks of the gene pairs under each seed measure in assenting order.

**Table 1:** Illustrative example for integration similarity.

Gene Pairs	semantic Similarity			Rank of Similarity			Integration of Ranks			
			
	*M_A_*	*M_B_*	*M_C_*	*M_A_*	*M_B_*	*M_C_*	MAX	MIN	MEAN	MEDIAN
Gene pair 1	2.4	0.2	0.04	9	2	4	0.9	0.2	0.5	0.4
Gene pair 2	1.8	0.6	0.12	6	7	8	0.8	0.6	0.7	0.7
Gene pair 3	3.0	0.9	0.03	10	10	3	1.0	0.3	0.8	1.0
Gene pair 4	1.2	0.3	0.05	5	3	5	0.5	0.3	0.4	0.5
Gene pair 5	0.9	0.1	0.06	3	1	6	0.6	0.1	0.3	0.3
Gene pair 6	0.5	0.5	0.02	2	6	2	0.6	0.2	0.3	0.2
Gene pair 7	1.0	0.4	0.09	4	4	7	0.7	0.4	0.5	0.4
Gene pair 8	1.8	0.4	0.13	6	4	9	0.9	0.4	0.6	0.6
Gene pair 9	0.2	0.7	0.01	1	8	1	0.8	0.1	0.3	0.1
Gene pair 10	2.1	0.8	0.16	8	9	10	1.0	0.8	0.9	0.9

Illustrative example for integration similarity, where *M_A_*, *M_B _*and *M_C _*are three seed gene-to-gene functional similarity measures.

### Adaptive integration approach

The rank-based semantic similarities of gene pairs from every seed measure provide an unique opportunity to compute the gene-to-gene similarities with all the information of GO utilized by the seed measures. A key problem here is how to select the most appropriate integration approach. Although there are many integration approaches all working well on certain domains, there does not exist one method that is always better than the others. In fact, to choose an appropriate integration method is largely dependent on the content of the study. Therefore, we propose an adaptive approach to automatically select the most appropriate integration method from a set of candidates. The main idea of the adaptive approach is to score all of the methods in the pool of the candidate integration approaches with the background set *BG*, and then select the best one.

InteGO provides four integration methods: max, min, mean and median. As an open system, InteGO also allows users to use their own integration methods. Mathematically, let *RankSim*(*g*1, *g*2, *m*) be rank-based similarity of gene *g*1 and *g*2 using seed measure *m*, InteGO is defined as:

(6) $$InteGO\left(g_{1},g_{2},I\right)=\left\{\begin{array}{c} max_{m\in M}RankSim\left(g_{1},g_{2},m\right)\;\;\;\;\;if\;I=max\\ min_{m\in M}RankSim\left(g_{1},g_{2},m\right)\;\;\;\;\;if\;I=min\\ mean_{m\in M}RankSim\left(g_{1},g_{2},m\right)\;\;\;\;if\;I=mean\\ median_{m\in M}RankSim\left(g_{1},g_{2},m\right)\;\;\;if\;I=median\\ integration_{m\in M}RankSim\left(g_{1},g_{2},m\right)\;if\;I=other\text{\_}integration \end{array}\right)$$

where *M *is a set of seed measures and *I *is an integration method which is max, min, mean, median of all of the ranks, or any other integration method that is defined by the user. For an illustrative example in Table 1, the results based on the four different integration methods are shown in the third column.

To automatically determine which integration method is the best, all of the gene pair similarities in *BG *are calculated based on each candidate integration method and are evaluated systematically with biological data. Recent studies used the correlation coefficient of gene expression correlations or gene sequence similarities to evaluate the MF based gene similarities [22]. However, it is not always correlated between gene functional similarities and gene expression correlation or sequence similarities [12]. Furthermore, previous studies show that enzymes are usually categorized biochemically with EC (Enzyme Commission) numbers but not their nucleotide or amino acid sequences [27,28], which indicates that it could be a better way for using EC numbers to explain molecular function with the criteria that the molecular functions of a group of genes are similar if they have the same EC numbers [12,29,30].

To systematically use EC to choose an integration method, all of the genes in *BG *are grouped based on their EC numbers (four digits), and then the differences between the inter- and intra-EC gene-to-gene similarities are tested. With an integration method, the higher the ratio between intra-EC gene similarities and inter-EC gene similarities, the better the integration method is. Quantitatively, we utilize the logged fold change (LogFC) measure which has been widely used in the gene expression studies [31]. The LogFC score of EC *ei *is defined in Eq 7:

(7) $$LogFC\left(e_{i}\right)=\frac{1}{\left|E\right|}\times \underset{e_{j}\in E;G\left(e_{j}\right)\cap G\left(e_{i}\right)=\theta }{\sum }\frac{\underset{g\in G\left(e_{i}\right)}{\sum }diff_{g}\left(e_{i},e_{j}\right)}{\left|G\left(e_{i}\right)\right|}$$

where *G*(*e_i_*) is set of all of genes which EC number is *e_i_*; *E *is a set of ECs which do not have overlapped genes with *e_i _*(*G*(*e_j_*) *∩ G*(*e_i_*) = ∅); *diff_g_*(*e_i_*, *e_j_*) is computed as:

(8) $$diff_{g}\left(e_{i},e_{j}\right)=\text{ln}\frac{|G\left(e_{i}\right)|\;\times \;\underset{g^{\prime }\in G\left(e_{j}\right)}{\sum }\left(1-GeneSim\left(g,g^{\prime }\right)+c\right)}{|G\left(e_{j}\right)|\;\times \;\underset{g*\in G\left(e_{i}\right)}{\sum }\left(1-GeneSim\left(g,g*\right)+c\right)}$$

where *c *is a Laplacian smoothing parameter which is a constant small positive number; *G*(*e_i_*) is the set of all of the genes assigned to EC *e_i _*except gene *g*; *G*(*e_j_*) is the set of all of the genes assigned to EC *e_j_*; *g *is a gene assigned to *e_i_*. In Eq 8, the numerator represents the inter-EC distance and the denominator represents the intra-EC distance. The higher the *diff_g_*(*e_i_*, *e_j_*) is, the more obvious the positive difference between inter-EC difference and intra-EC difference is.

For example, given nine genes in *BG*, four of which have the same EC number, labeled as *e*_1_, and the other five genes belong to another EC number, labeled as *e*_2_. To calculate the LogFC score for *e*_1_, we first compute *diff_g_*(*e*_1_, *e*_2_) with Eq 8, meaning that every gene in *e*_1 _is compared with every other gene in *e*_1 _for the average intra-EC difference, and then every gene in *e*1 is compared with every gene in *e*_2 _to get the inter-EC differences. *logFC*(*e*_1_) is the average of all of the *diff_g_*(*e*_1_, *e*_2_) scores for the genes assigned to *e*_1_.

The method that has the highest LogFC scores for all of the ECs are considered as the most appropriate integration method for *BG*. If a user input set *G *is much smaller than *BG *(which often happens), we assume the selected method is also the most suitable for *G *∪ *BG*. If the size of *G *is comparable to *BG*, it is not necessary to use *BG*, then the integration method shall be selected based on the evaluation on *G*.

## Results

To systematically evaluate the performance of InteGO, we tested it on three model organisms with different levels of GO annotation scale and complexity. For each of them, we adopted EC numbers and protein sequences as independent biological evidences.

### Data preparation

The GO annotation and structure data were downloaded from the GO website (http://www.geneontology.org/GO.downloads.shtml). To systematically evaluate different GO-based gene-to-gene similarity measures on MF, the pathway and EC number information of Yeast, *Arabidopsis *were downloaded from the Saccharomyces genome database (http://biocyc.org/YEAST/organism-summary?object=YEAST), PlantCyc (http://ftp.plantcyc.org/Pathways) and HumanCyc (http://humancyc.org) respectively. Note that our EC based evaluation method requires that an EC has at least two genes. In yeast, *Arabidopsis *and human, 95, 325 and 312 ECs satisfy the criteria. The protein sequences were downloaded from the Saccharomyces genome database (http://www.yeastgenome.org/download-data/sequence), TAIR (http://www.arabidopsis.org/tools/bulk/sequences/index.jsp) and UniProt (http://www.uniprot.org) respectively.

Let *E *be the set of all of the ECs that have at least one gene assignment, we define *BG *as the set of all of the genes that has at least one EC assignments in *E*. This definition of *BG *ensures that for any gene in *BG *the intra-EC similarity is valid. The sizes of *BG *are 218, 1,348 and 1,504 for yeast, *Arabidopsis *and human respectively. An experiment on the variation of the background set (see Additional file 1) reveals that the use of a relatively smaller background set may affect performance significantly. Additional file 2, 3 and 4 show that the distribution of the gene-to-gene similarities with Yu, Schlicker and Wang measures, where the similarity scores are spread in the full spectrum of the range. In summary, the background gene sets are well prepared.

InteGO was implemented with Java JDK 1.6 and JUNG library [32]. The experiments were run on a windows 7 computer with Intel i7 CPU and 10 GB RAM.

### Selecting seed measures

**Figure 3:**
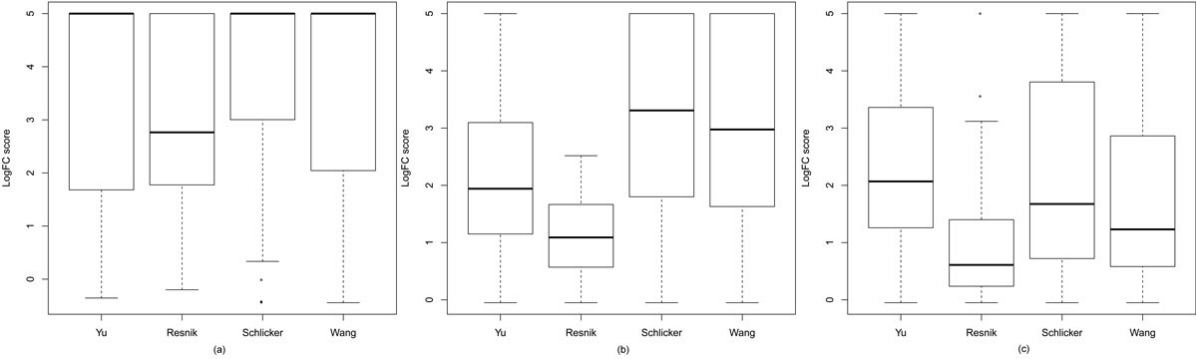
**Logged fold change (LogFC) score comparison**. Logged fold change (LogFC) score comparison for four different similarity measures in Molecular Function (MF) category on yeast (a), *Arabidopsis*(b) and human (c).

In order to select the most appropriate seed measures for InteGO, we screened four existing measures (Yu, Resnik, Schlicker and Wang) using the EC based evaluation method. Figure 3 shows that for the Yu, Schlicker and Wang measures, it is not distinguishable that one measure is significant better than another. The Yu, Schlicker and Wang measures all performed the best on yeast with the highest median value. The Schlicker measure performs best on *Arabidopsis*, while the Yu measure is best on human. Therefore, we chose all of the three as the seed measures in InteGO. We did not choose the Resnik measure, because it is clearly not as good as the other measures in all of the three species. Note that the upper-bound and the lower-bound of the LogFC scores in Figure 3 were set to 5 and -0.05 respectively to eliminate outliers.

**Figure 4:**
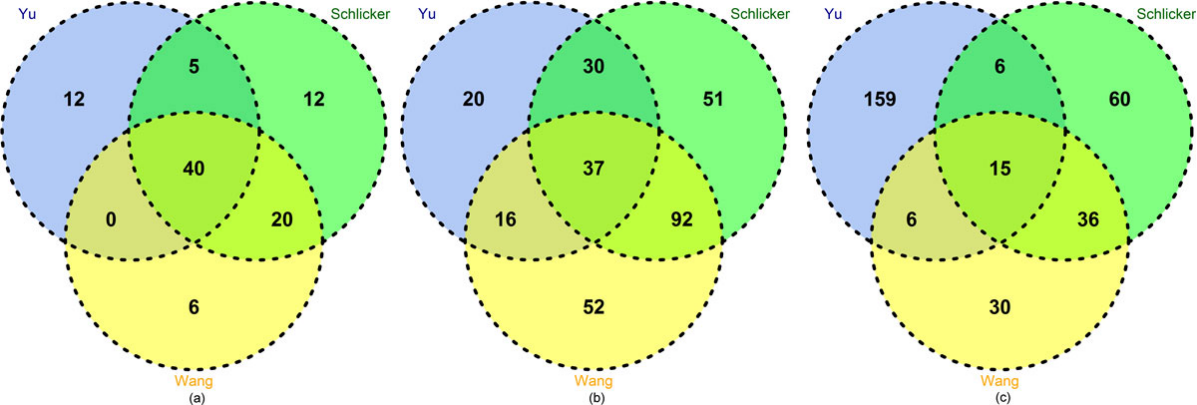
**Venn Diagram for Yu measure, Schlicker measure and Wang measure with number of ECs on which perform best on yeast (a), Arabidopsis (b) and human (c)**. Venn Diagram for Yu measure, Schlicker measure and Wang measure with number of ECs on which perform best on yeast (a), *Arabidopsis *(b) and human (c). Blue, green and yellow represent Yu measure, Schlicker measure and Wang measure respectively.

In addition, Figure 4 shows that although all of the three seed measures perform equally well in some ECs, each measure has its own favorable EC groups. For example, the Schlicker and Wang measures perform the best in 51 and 52 out of the total 325 *Arabidopsis *ECs respectively (see Figure 4(b)), which is greater than the Yu measure (20 ECs). However, the Yu measure performs the best in 159 out of the total 315 human ECs, which dominant the EC group distribution in human (see Figure 4(c)). Therefore, an appropriate integration of these measures may combine the advantages of different measures and improve the overall performance. Note that although only four measures were screened in the experiment, more measures can be evaluated and added later since InteGO has an open framework.

### Selecting integration method

**Figure 5:**
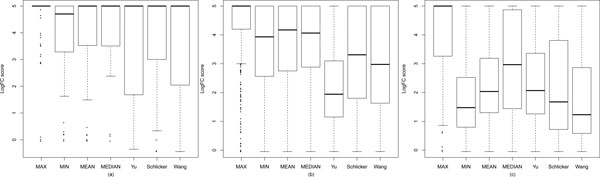
**Logged fold change (LogFC) score comparison for four integration measures and three integrated measures**. Logged fold change (LogFC) score comparison for four integration measures (MAX, MIN, MEAN and MEDIAN) and three integrated measures (Yu measure, Schlicker measure and Wang measure) in Molecular Function (MF) category on yeast (a), *Arabidopsis *(b) and human (c).

In order to select the most appropriate integration method, four different approaches (MAX, MIN, MEAN and MEDIAN) were tested and compared. Figure 5 shows that MAX performs the best among the four integration methods. In yeast, although almost all of the measures have the same median value, the 25th percentile of MAX is 5, significantly higher than the Yu, Schlicker and Wang measure (1.68, 3.00 and 2.04 respectively) and the other integration methods. In *Arabidopsis *and human, the median of MAX are both 5, which is also significantly higher than that of all of the other integration methods. It indicates that the performance of MAX, a simple integration approach, has been increased to around 2-fold. This is because the integration considers all of the aspects of GO, while an individual seed measure, although nicely designed, is compromised in that it focuses on only one of few kinds of knowledge in GO. The other integration measures, especially MIN, however, cannot distinguishably improve the gene similarity performance. As shown in Figure 5(c), the result of MIN is even worse than the seed measures. It indicates that the performance of gene-to-gene similarity could be significantly improved only by the appropriate integration.

**Figure 6:**
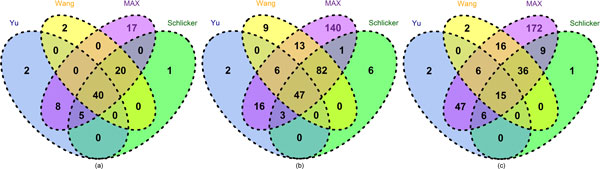
**Venn Diagram for the best integration measure MAX and Yu measure, Schlicker measure and Wang measure**. Venn Diagram for the best integration measure MAX and Yu measure, Schlicker measure and Wang measure with number of ECs on which perform best on yeast (a), *Arabidopsis *(b) and human (c). Purple, blue, green and yellow represent MAX measure, Yu measure, Schlicker measure and Wang measure respectively.

As mentioned in the previous section, the seed measures have their own favorable EC groups. To test whether MAX take advantage of all of the strength of the seed measures, we compared MAX with the Yu, Schlicker and Wang measure on all of the ECs. Figure 6 (a), (b) and (c) show that MAX dominant the EC groups, clearly different to the results in Figure 4. In detail, MAX performs the best in 140 and 172 out of 325 and 315 ECs in *Arabidopsis *and human respectively, while the numbers are only 2, 9, 6 in *Arabidopsis *and 2, 2, 1 in human for the Yu, Wang and Schlicker measures respectively. In summary, the experiment indicates that integrating multiple measures could improve the performance of gene similarity measurement and MAX is the best integration method.

Statistics analysis was carried out to test whether the results of the best integration measure (MAX) of InteGO is statistically the best. We compared InteGO with the three seed measures using TukeyHSD test [33]. The p-values shown in Table 2 and the 95% family-wise confidence level (Additional file 5, 6 and 7) indicate that the results of MAX are significant better than the results of all of the seed measures in yeast, *Arabidopsis *and human, with the only exception that the Schlicker measure's results are comparable in yeast, in that the Schlicker measure performs very well in yeast, so there is little room for InteGO to improve.

**Table 2:** Adjusted P-values for comparing MAX with Yu, Schlicker and Wang measure using TukeyHSD.

Measures	Adjusted p-value		
	
	yeast	*Arabidopsis*	human
MAX vs. Schlicker	2.8E-1	*<***1.0E-7**	*<***1.0E-7**
MAX vs. Wang	**1.0E-2**	*<***1.0E-7**	*<***1.0E-7**
MAX vs. Yu	**1.1E-4**	*<***1.0E-7**	*<***1.0E-7**

Wang vs. Schlicker	5.9E-1	9.6E-1	3.2E-1
Yu vs. Schlicker	6.0E-2	*<***1.0E-7**	1.9E-1
Yu vs. Wang	5.8E-1	*<***1.0E-7**	**1.2E-3**

Adjusted P-values for comparing MAX with Yu, Schlicker and Wang measure using TukeyHSD. Significant p-values are in bold fonts.

### Protein sequence based performance evaluation

**Figure 7:**
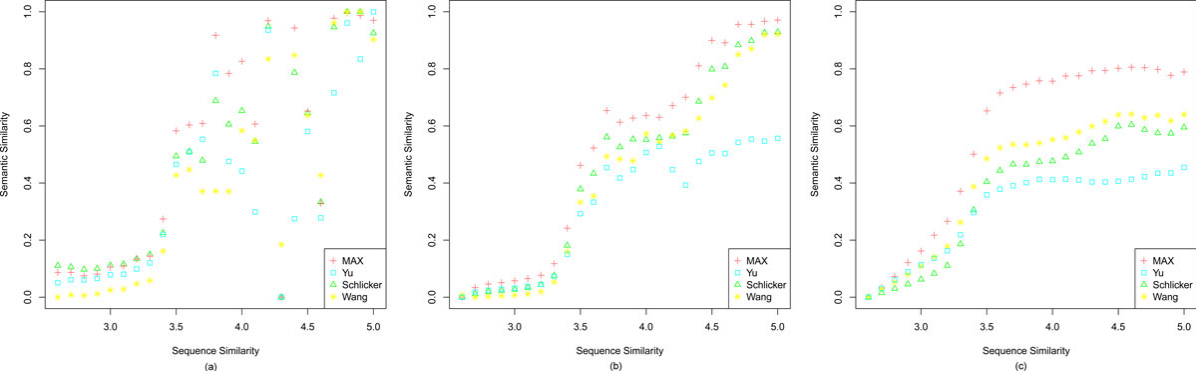
**Comparing InteGO with the Yu, Schlicker and Wang measures with protein sequence similarity**. Comparing InteGO with the Yu, Schlicker and Wang measures with protein sequence similarity on yeast (a), *Arabidopsis *(b) and human (c), where the x-axis is BLAST sequence similarity (*ln*(*BitScore*)) and y-axis is the normalized semantic similarity based on GO.

In addition to use EC as the evaluation criteria, protein sequence similarities were employed as independent evidence for further performance study. Although the correlation coefficient between semantic similarity and sequence similarity is not as strong as EC, it is generally accepted that as sequence similarity increases, so does the chance that these proteins are homologues, in which case they are likely to have identically annotated molecular functions [34]. In our test, sequence similarity scores (*ln*(*BitScore*)) of all genes in the *BG *of the three species were calculated with BLAST, resulting in 20,652 yeast, 772,609 *Arabidopsis *and 942,609 human gene pairs. As shown in Figure 7, the semantic similarity measurements show a correlation with sequence similarity. The covariance scores (see Additional file 8) on all of the three species reveal that InteGO is overall the best measure.

## Conclusions

Comparing gene at the functional level is vital for various of applications [3-7]. The existing GO semantic based measures either calculate gene-to-gene similarities directly [13], or indirectly compute gene-to-gene similarities with term-to-term similarities [12,17]. Unfortunately, none of them takes into account all of the respects of rich information in GO (structure, annotation, LCA and all of the parents term, *etc*). In this paper, we proposed a new measure called InteGO to appropriately integrate the seed measures with the following advantages: 1) InteGO employs an adaptive approach which enables the optimization of seed measure integration; 2) it applies a rank-based integration approach, which unifies the scale and distribution differences among different seed measures; 3) InteGO is an open-platform measure that allows users to add/delete seed measures, redefine the background gene set and change the rank-based integration method.

To demonstrate the advantages of InteGO, we compared its EC-assigned gene similarities and sequence similarities with three existing measures (the Yu, Schlicker and Wang measure) in yeast, *Arabidopsis *and human. Comparing with these state-of-the-art measures, the experimental results show that InteGO increases the LogFC scores to about two-fold. It indicates that integrating multiple measures appropriately can improve the performance of the functional similarity measure. Especially, we found that taking the maximal ranks from all of the seed measures performs the best. The covariances between semantic similarities and protein sequence similarities shows InteGO is clear the best out of all the tested measures.

In InteGO, to maintain a large background gene set is expensive. Therefore, extending InteGO from MF to BP or even other biological or medical ontologies is not a trivial problem. In the future, we will continue to improve InteGO to be more efficient and to be applicable on more ontologies. As an open framework, the performance of InteGO may be further improved by synergistically integrating more seed measurements. We will continue to integrate and compare InteGO with more recent gene-to-gene measurements in the future. We will continue to explore better integration methods, such as using EM algorithm to optimize the weight for each seed measure, to achieve better performance.

## Competing interests

The authors declare that they have no competing interests.

## Authors' contributions

**JC **conceived the project. **JP**, **JC **and **YW **designed the algorithm and experiments. **JP **implemented the algorithm and finished the experiments.

## Supplementary Material

Additional file 1 **Average LogFC scores for different sizes of background set**. To test whether the selection of *BG *will affect the integration performance, we compared the results for different background set on yeast. First, given the full set of *BG*, a subset of gene pairs were randomly selected with the percentage varying from 10% to 100%. This process was repeated for 100 times. Second, as shown in Additional file 1, the logFC scores for each subset size were calculated based on the randomly selected gene pairs. Since we do not use the full set, the computable ECs are also a subset of all of the computable ECs. In Additional file 1, the LogFC score increases linearly from 0 to 10 when the coverage increases from 10% to 90%, then suddenly jumps to a high score (13.8) when all of the background genes were used, indicating that first, the size of the background set affects the integration measure significantly, second, to use the full background set is the best, although it slightly increases the computational time. Click here for file

Additional file 2 **Distribution of the gene-to-gene similarities with Yu measure**. Distribution of the gene-to-gene similarities with Yu measure for all of the genes in the Background Gene Set (BG) on yeast. Click here for file

Additional file 3 **Distribution of the gene-to-gene similarities with Schlicker measure**. Distribution of the gene-to-gene similarities with Schlicker measure for all of the genes in the Background Gene Set (BG) on yeast. Click here for file

Additional file 4 **Distribution of the gene-to-gene similarities with Wang measure**. Distribution of the gene-to-gene similarities with Wang measure for all of the genes in the Background Gene Set (BG) on yeast. Click here for file

Additional file 5 **The 95% family-wise confidence level of TukeyHSD test on yeast**. The 95% family-wise confidence level of TukeyHSD test on yeast, which compared MAX with all the three seed measures (Schlicker, Wang and Yu measure). Click here for file

Additional file 6 **The 95% family-wise confidence level of TukeyHSD test on Arabidopsis**. The 95% family-wise confidence level of TukeyHSD test on *Arabidopsis*, which compared MAX with all the three seed measures (Schlicker, Wang and Yu measure). Click here for file

Additional file 7 **The 95% family-wise confidence level of TukeyHSD test on human**. The 95% family-wise confidence level of TukeyHSD test on human, which compared MAX with all the three seed measures (Schlicker, Wang and Yu measure). Click here for file

Additional file 8 **The covariance sores comparing with sequence similarity**. The covariance sores comparing with sequence similarity on yeast, *Arabidopsis *and human for Max, Yu, Schlicker and Wang measure. Click here for file

## Declarations

The publication costs for this article were funded by the corresponding author's institution.

This article has been published as part of *BMC Bioinformatics *Volume 15 Supplement 2, 2014: Selected articles from the Twelfth Asia Pacific Bioinformatics Conference (APBC 2014): Bioinformatics. The full contents of the supplement are available online at http://www.biomedcentral.com/bmcbioinformatics/supplements/15/S2.
