# Supplementary figures and images for: Towards integrative gene functional similarity measurement
Source: BMC Bioinformatics. 2014 Jan 24;15(Suppl 2):S5. doi: 10.1186/1471-2105-15-S2-S5 (PMC4015993; doi:10.1186/1471-2105-15-S2-S5)

Average of LogFC scores

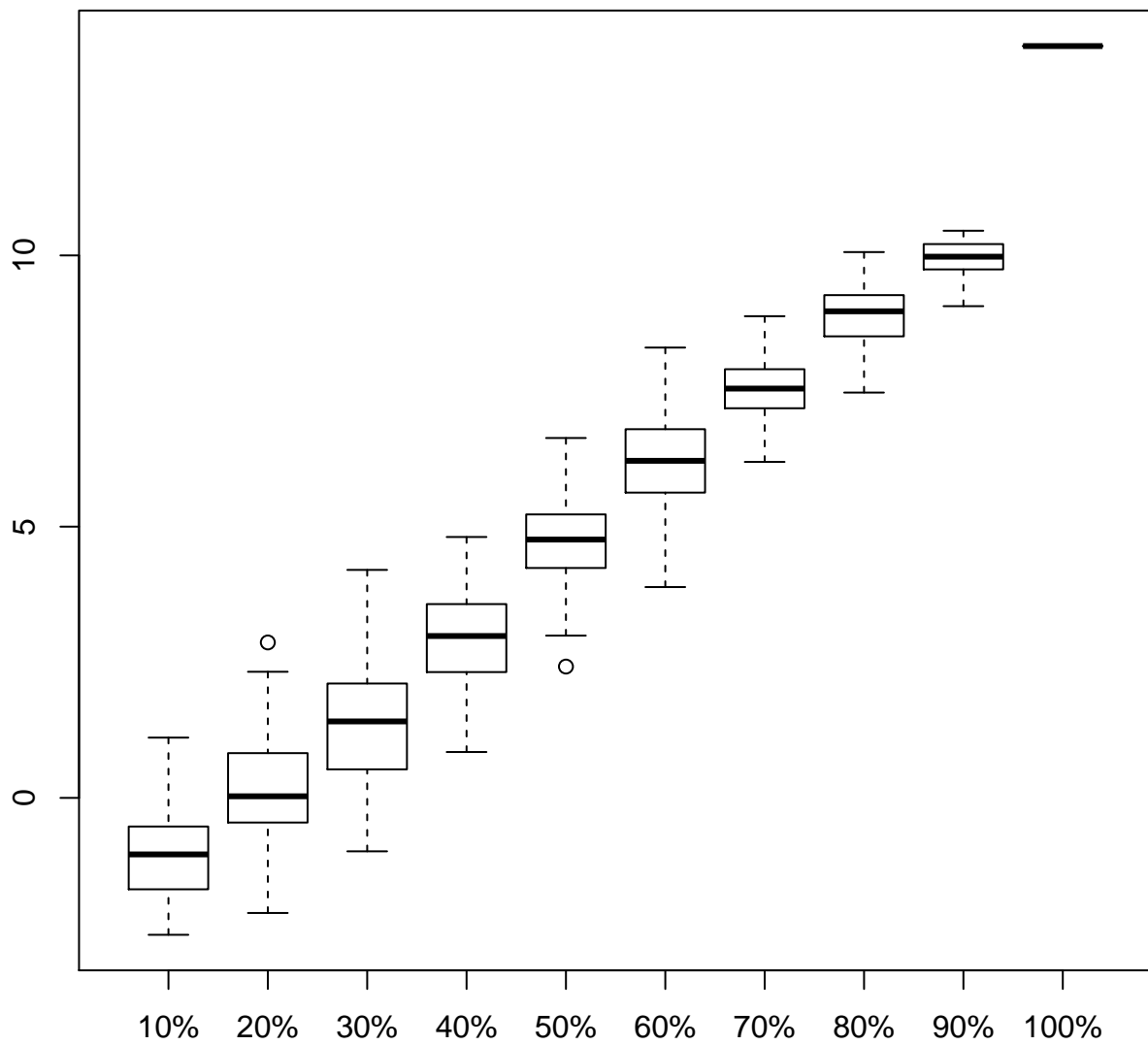

Supplement: Additional file 1 — Average LogFC scores for different sizes of background set. To test whether the selection of BG will affect the integration performance, we compared the results for different background set on yeast. First, given the full set of BG, a subset of gene pairs were randomly selected with the percentage varying from 10% to 100%. This process was repeated for 100 times. Second, as shown in Additional file 1, the logFC scores for each subset size were calculated based on the randomly selected gene pairs. Since we do not use the full set, the computable ECs are also a subset of all of the computable ECs. In Additional file 1, the LogFC score increases linearly from 0 to 10 when the coverage increases from 10% to 90%, then suddenly jumps to a high score (13.8) when all of the background genes were used, indicating that first, the size of the background set affects the integration measure significantly, second, to use the full background set is the best, although it slightly increases the computational time. [file 1471-2105-15-S2-S5-S1.pdf]

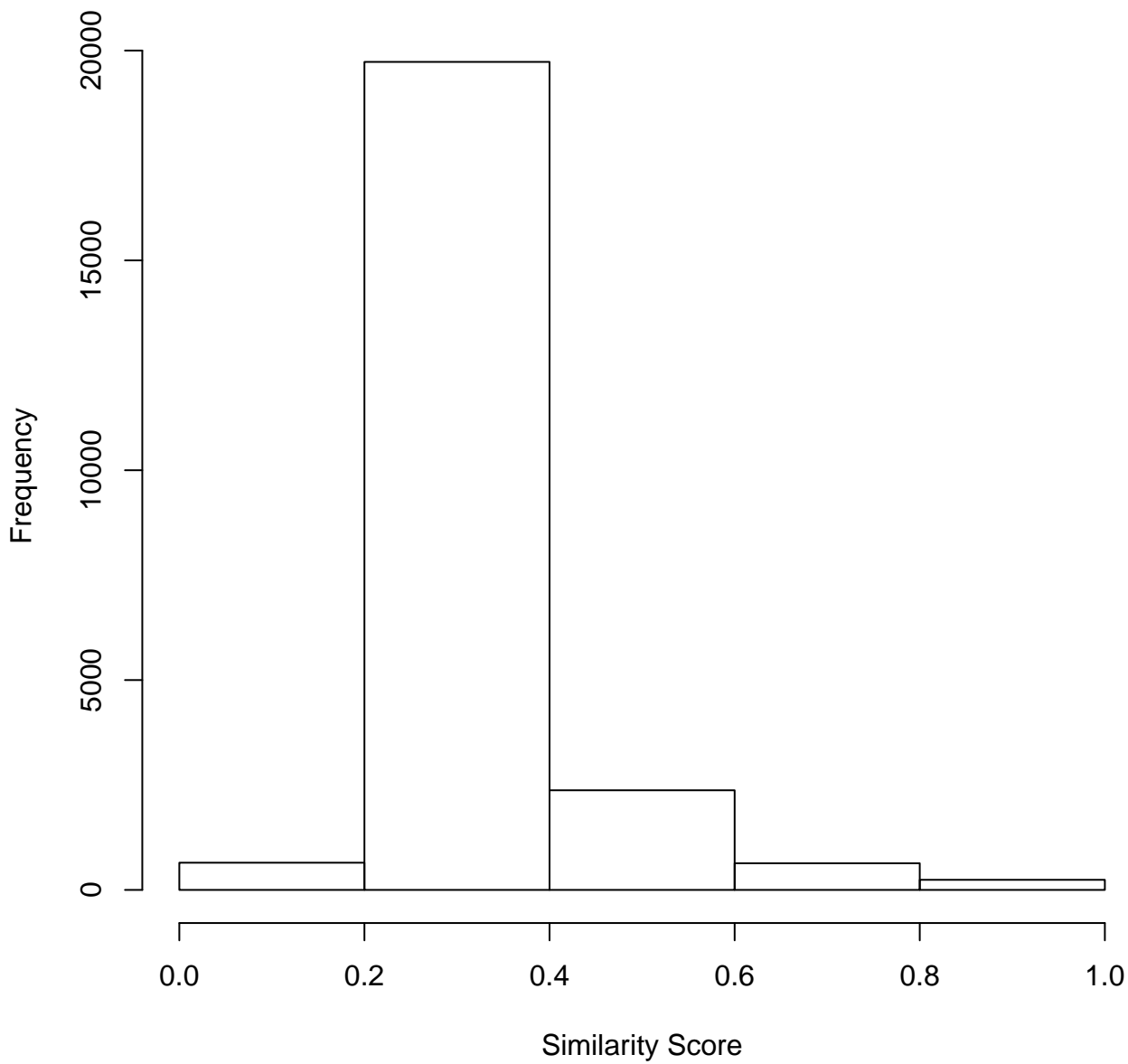

Supplement: Additional file 2 — Distribution of the gene-to-gene similarities with Yu measure. Distribution of the gene-to-gene similarities with Yu measure for all of the genes in the Background Gene Set (BG) on yeast. [file 1471-2105-15-S2-S5-S2.pdf]

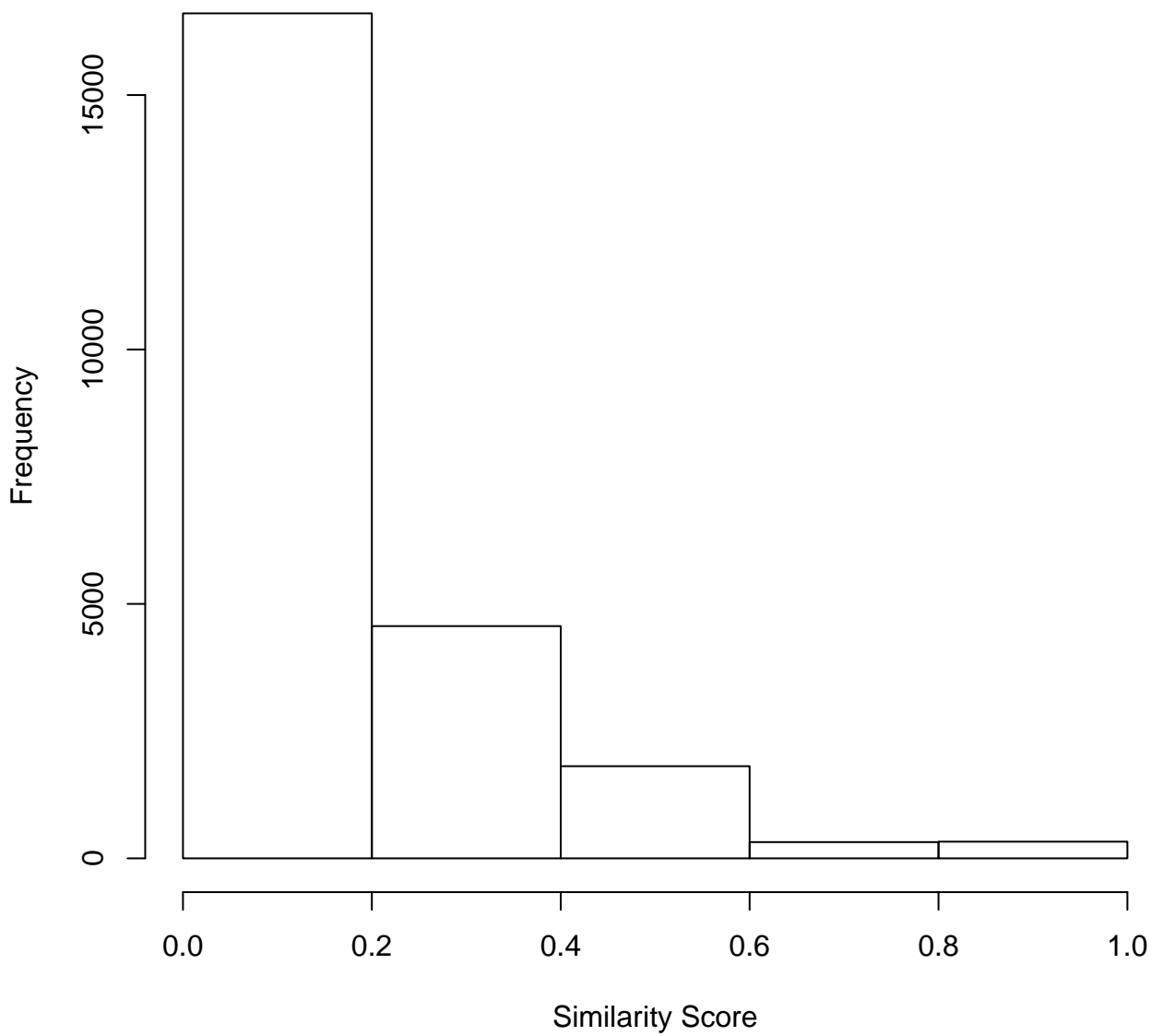

Supplement: Additional file 3 — Distribution of the gene-to-gene similarities with Schlicker measure. Distribution of the gene-to-gene similarities with Schlicker measure for all of the genes in the Background Gene Set (BG) on yeast. [file 1471-2105-15-S2-S5-S3.pdf]

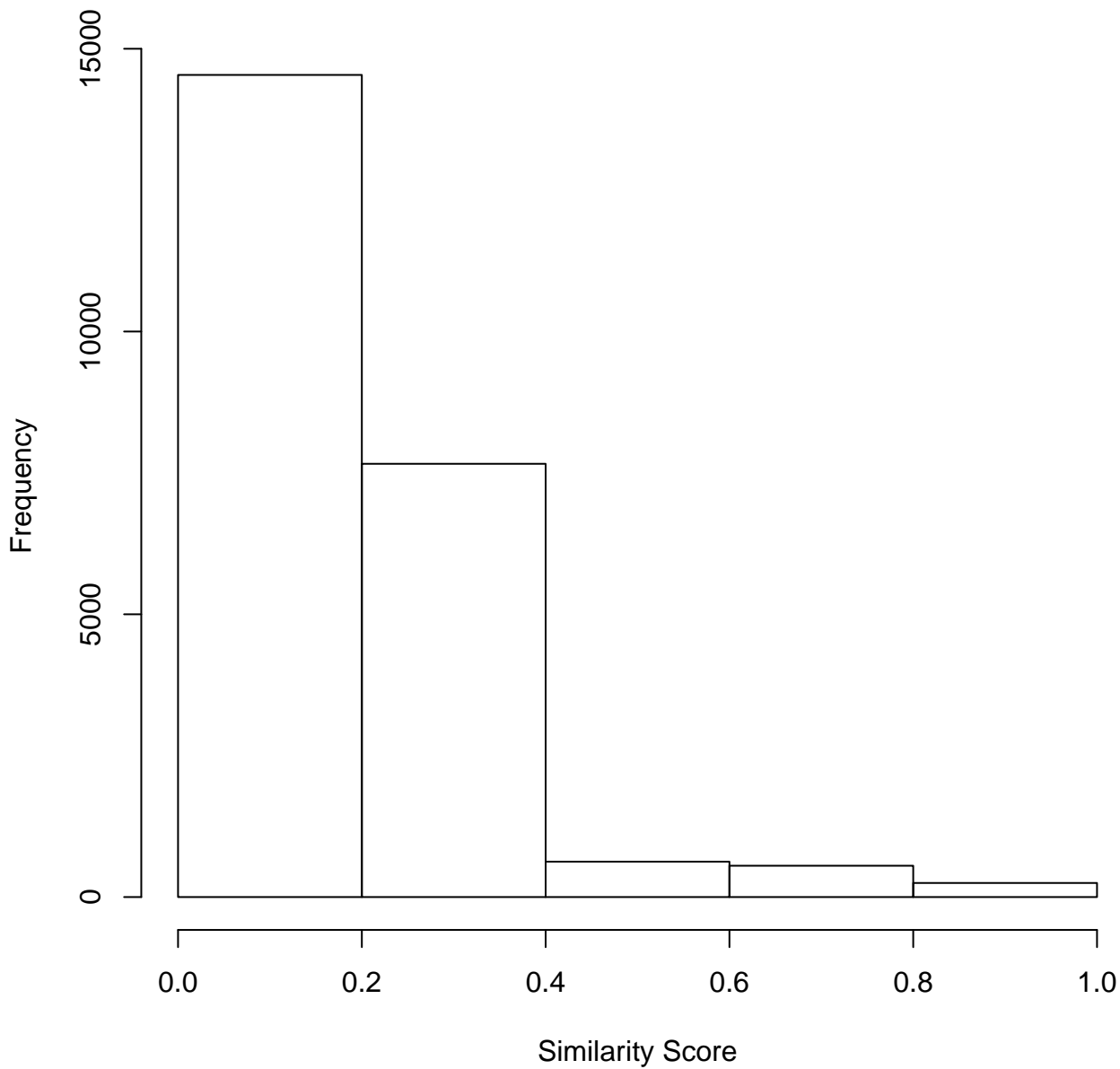

Supplement: Additional file 4 — Distribution of the gene-to-gene similarities with Wang measure. Distribution of the gene-to-gene similarities with Wang measure for all of the genes in the Background Gene Set (BG) on yeast. [file 1471-2105-15-S2-S5-S4.pdf]

# 95% family-wise confidence level

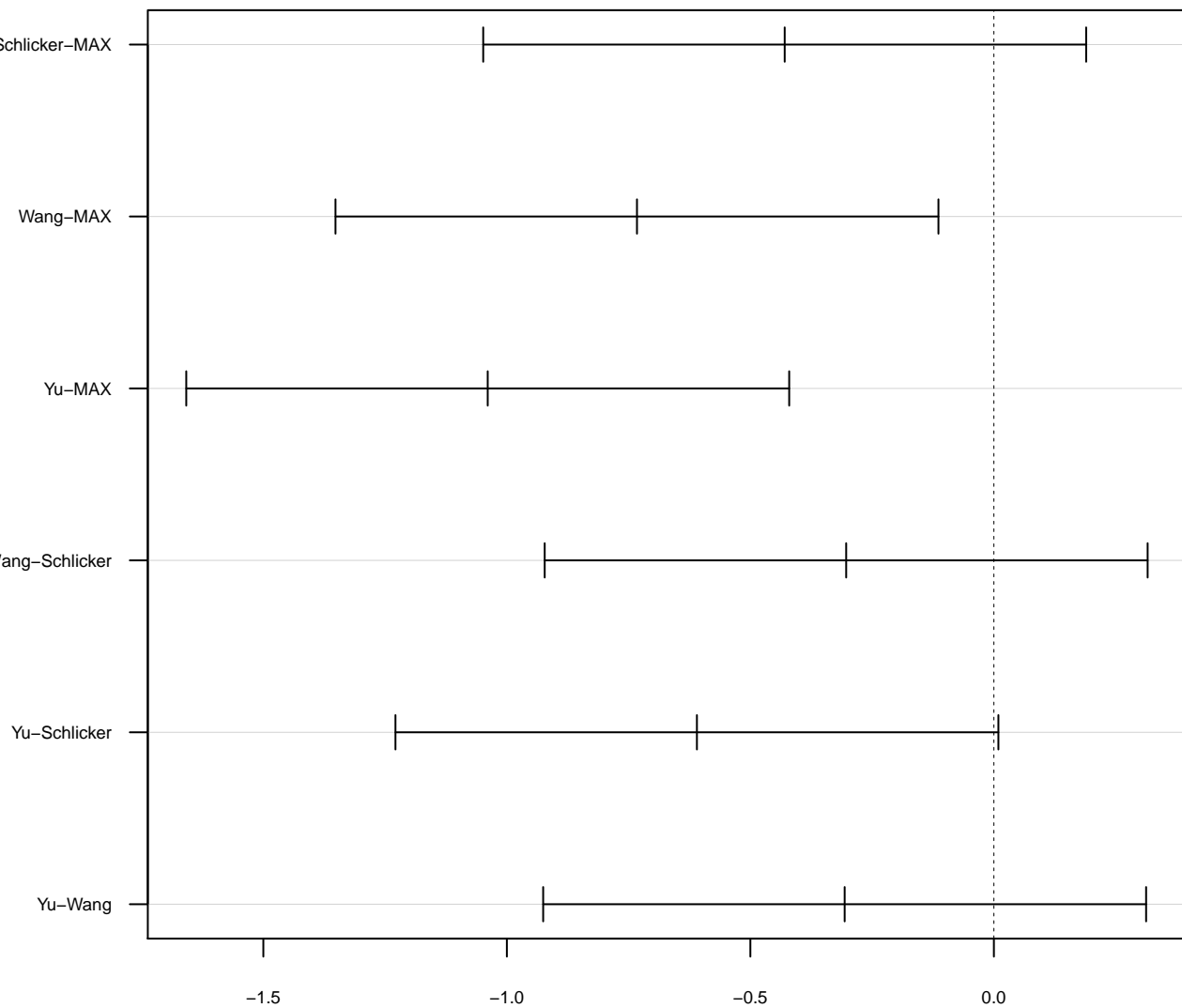

Differences in mean levels of Measure

Supplement: Additional file 5 — The 95% family-wise confidence level of TukeyHSD test on yeast. The 95% family-wise confidence level of TukeyHSD test on yeast, which compared MAX with all the three seed measures (Schlicker, Wang and Yu measure). [file 1471-2105-15-S2-S5-S5.pdf]

# 95% family-wise confidence level

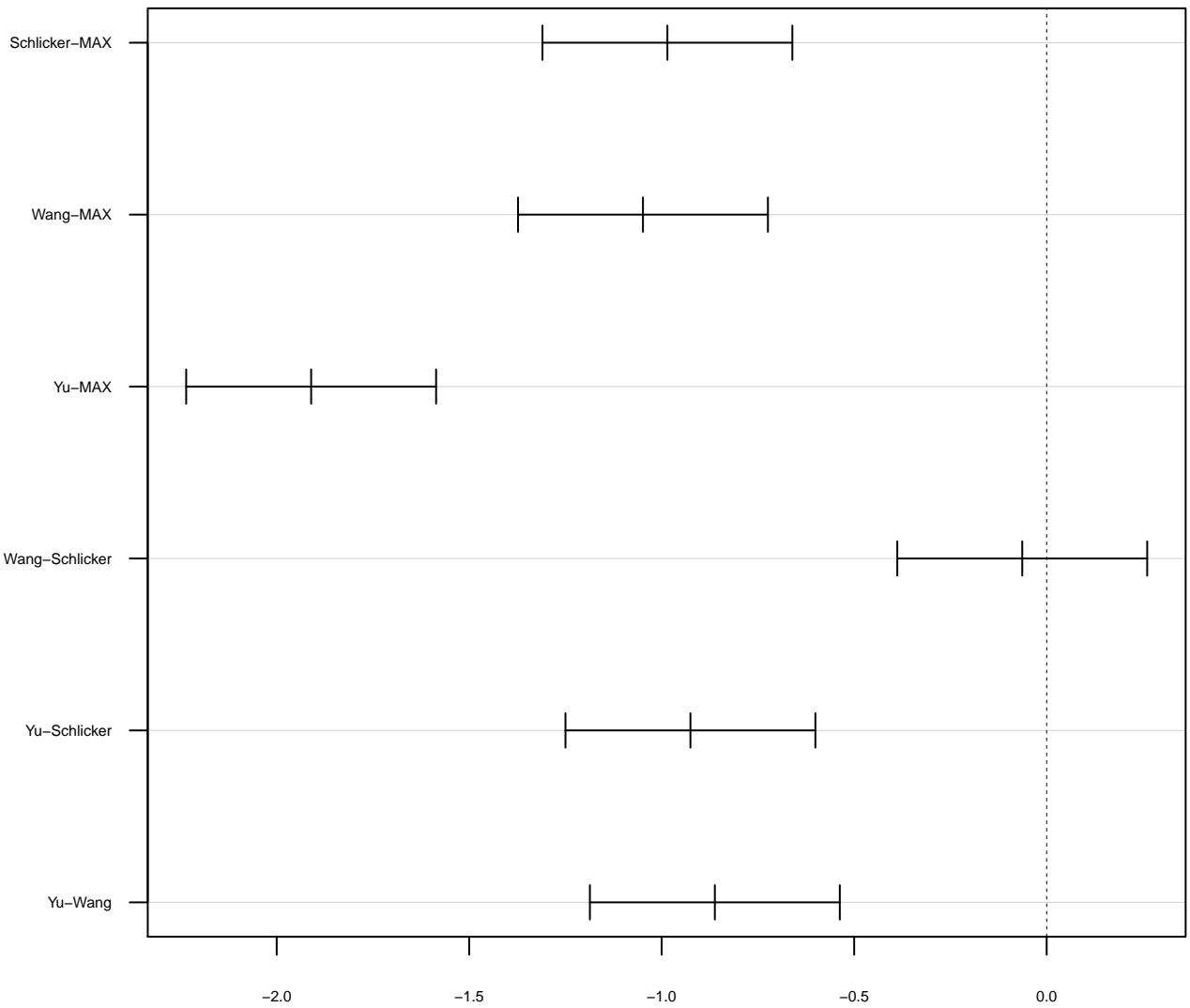

Differences in mean levels of Measure

Supplement: Additional file 6 — The 95% family-wise confidence level of TukeyHSD test on Arabidopsis. The 95% family-wise confidence level of TukeyHSD test on Arabidopsis, which compared MAX with all the three seed measures (Schlicker, Wang and Yu measure). [file 1471-2105-15-S2-S5-S6.pdf]

# 95% family-wise confidence level

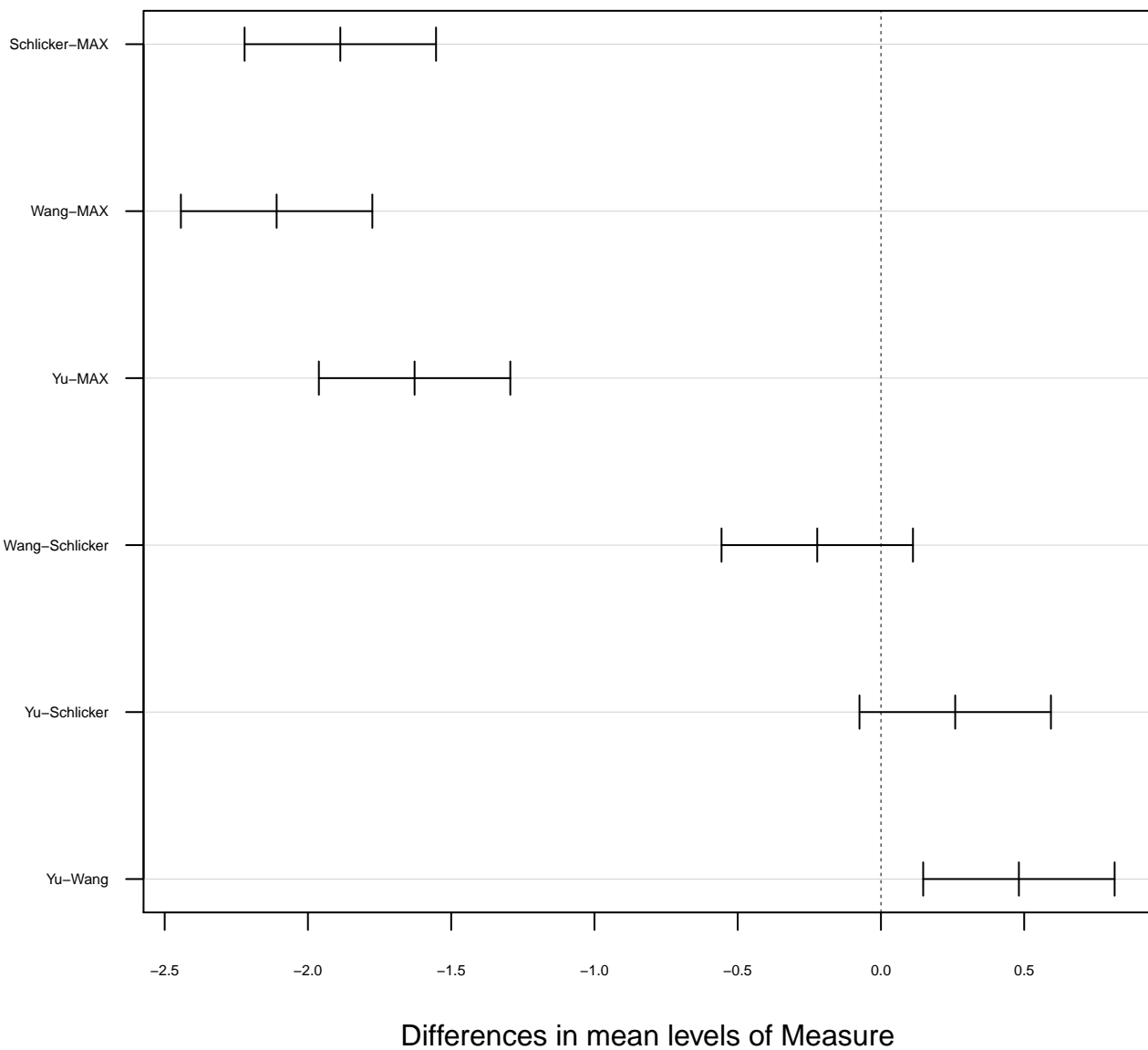

Supplement: Additional file 7 — The 95% family-wise confidence level of TukeyHSD test on human. The 95% family-wise confidence level of TukeyHSD test on human, which compared MAX with all the three seed measures (Schlicker, Wang and Yu measure). [file 1471-2105-15-S2-S5-S7.pdf]
